# HPV infection - Screening, diagnosis and management of HPV-induced lesions: Number 3 - March 2021

**DOI:** 10.1055/s-0041-1727285

**Published:** 2021-04-15

**Authors:** Ana Katherine da Silveira Gonçalves de Oliveira, Claudia Marcia de Azevedo Jacyntho, Fernanda Kesselring Tso, Neide Aparecida Tosato Boldrini, Neila Maria de Góis Speck, Raquel Autran Coelho Peixoto, Rita Maira Zanine, Yara Lucia Mendes Furtado de Melo

**Affiliations:** 1 Universidade Federal do Rio Grande do Norte, Natal, RN, Brazil.; 2 Hospital Federal dos Servidores do Estado do Rio de Janeiro, Rio de Janeiro, RJ, Brazil.; 3 Escola Paulista de Medicina, Universidade Federal de São Paulo, São Paulo, SP, Brazil.; 4 Universidade Federal do Espírito Santo, Vitória, ES, Brazil.; 5 Escola Paulista de Medicina, Universidade Federal de São Paulo, São Paulo, SP, Brazil.; 6 Universidade Federal do Ceará, Fortaleza, CE, Brazil.; 7 Universidade Federal do Paraná, Curitiba, PR, Brazil.; 8 Universidade Federal do Rio de Janeiro, Rio de Janeiro, RJ, Brazil.; 9 Universidade Federal do Estado do Rio de Janeiro, Rio de Janeiro, RJ, Brazil.

## Key points

- Address the importance of organized screening for cervical cancer.
- Indicate new technologies in screening.
- Anogenital warts are caused by HPV, mainly 6 and 11.
- The diagnosis of condyloma acuminata is clinical with several effective therapeutic options.
- Low-grade squamous intraepithelial lesion (LSIL) and atypias of undetermined significance in squamous cells (ASC-US).
- As the risk of histological high-grade squamous intraepithelial lesion (HSIL) in a patient with cytological LSIL is significant, the quality of the cytopathological examination is essential.
- Cases of cytological HSIL should be systematically referred to colposcopy.
- “See-and-treat” is a diagnostic and therapeutic procedure and must be performed according to rules established for non-pregnant women over 25 years of age.
- “See-and-treat” should not be performed in adolescents given the future possibility of obstetric comorbidities.

## Recommendations

- Screening should be organized based on population data records with wide coverage.
- The HPV DNA test must be offered for screening.
- The therapeutic method for warts should be changed if there is no significant improvement within four weeks of treatment.
- Podophyllin, 5-fluorouracil and imiquimod should not be used during pregnancy.
- Conservative therapy for HSIL is preferred in patients ≤ 25 years of age.
- In patients ≥ 25 years of age, risk estimates such as immunosuppression, HPV DNA positive and previous altered cytology and/or biopsy results should be considered.
- The cytopathology of HSIL should be referred to colposcopy, it is not acceptable to repeat the cytopathology exam.
- The endocervical canal should be evaluated with cytological brushing or curettage when the squamocolumnar junction (SCJ) is not visible.
- After treatment of HSIL, follow-up with cytology and colposcopy is recommended every six months for two years then, annual cytology until completing five years. If possible, perform HPV DNA test on high-risk patients after six months and annually for three years; if tests are negative, return to triennial screening for 25 years.

## Background

Human papillomavirus (HPV) is the most common sexually transmitted infection in the world. The risk of being infected with the virus at least once in your life is of 50%. 1 The most common oncogenic types are HPV-16 and 18, and their persistence is the main cause of female lower genital tract cancer. It is estimated that 33,369 cases of HPV-associated cancers are diagnosed each year in the USA, with 21,290 (13.2/100,000) cases in women. 1 More than 600,000 cancer cases worldwide are attributed to HPV annually, which is the most important risk factor in the development of cervical, vagina, vulva, penis, anus and oropharynx neoplasms, in addition to causing anogenital warts. 2

## Cervical cancer screening

Cervical cancer is a public health problem worldwide and the fourth most common type of tumor among women, with 500,000 new cases and 250,000 deaths per year. 3 In Brazil, it is the third most common cancer in women. According to the National Cancer Institute, estimates for 2020 are of 16,590 new cases; and 6,526 deaths occurred in 2018. 4 The main cause of the development of cervical cancer is persistent infection with oncogenic HPV types. Infections are more prevalent in adolescents and young adults, with peak prevalence in the early years of sexual activity. 5 Effective screening has shown a reduction in the incidence of this neoplasia and mortality due to it. Precursor lesions of cancer, when diagnosed and treated, prevent progression to invasive lesion. 6 Brazil still has an opportunistic form of screening. 5 The strategy that has been shown to be most effective is the organized population based screening program adopted by most European countries. This program advocates a system of active approach (call/recall system), based on the personal invitation of the target population. 6 The organized screening program has reached more women, showing high coverage of the territory. 3 5

## What is the target population for screening and how often should it be done?

According to Brazilian Guidelines for the Screening of Cervical Cancer (Ministry of Health - MH), 7 the screening method used is oncotic cytology from 25 years of age for women who have already started sexual life. Tests must continue until the age of 64, and in women with no previous history of pre-neoplastic disease, they should be stopped when they have had at least two negative tests in the last five years. 7 For women over 64 years of age who have never undergone the exam, two exams should be carried out every one to three years. If both tests are negative, these women may be excused from screening. The recommended periodicity is every three years after two negative exams with a one-year interval in between. Immunosuppressed women must undergo the screening test after the beginning of sexual activity with semiannual intervals in the first year, maintaining an annual follow-up afterwards. In HIV + women with a TCD4 + lymphocyte count below 200 cells/mm3, semiannual screening should be maintained. 7 The American Cancer Society (ACS – 2020), 8 like most European countries, recommends that screening should also start at 25 years of age and be suspended at 65 years of age; in Europe, the age ranges between 59 and 65 years. 6 The frequency varies from three to five years, depending on the method - cytology, HPV DNA test or co-test. 8

## What are the screening methods?

### Pap smear

The Brazilian Guidelines recommend the periodic performance of conventional cytology, although this is characterized by low reproducibility among observers. Liquid-based cytology (LBC), created with the aim of reducing unsatisfactory smears, is an alternative method of screening. Cells are deposited in a fixative suspension, which allows their uniform distribution on the slides after processing. Another advantage is the possibility to carry out new tests on the material residue in the liquid medium, such as HPV DNA test, although LBC has not shown a gain in sensitivity, compared to conventional smears. 9

### HPV DNA tests

Evidence of the causal relationship between infection by oncogenic HPV types and the onset of precursor lesions and cervical cancer favored the creation of new HPV DNA detection technologies. 10 Current evidence supports the use of the test in primary screening in women aged ≥ 30 years. Screening with this test can be performed every five years. Brazilian Guidelines recommend HPV DNA testing from the age of 30, extending it to 64 years of age. When positive for oncogenic types, the reflex cytological examination must be performed. 10 Another advantage of HPV DNA testing is the possibility of self-sampling, which is done by the woman herself anywhere. Detection rates of HPV in this form have been similar to clinician-collected rates. 5 The American Cancer Society recommends HPV DNA testing as a primary screening or in association with cytology, called a co-test, although this is already advocated as primary screening in future guidelines. 8 About 19 of 28 countries in Europe already track this way. 6

## What is Febrasgo's recommendation for cervical cancer screening in Brazilian women?

In 2018, Febrasgo published the dossier proposing strategies to add to the 2016 Brazilian Guidelines (Ministry of Health - National Cancer Institute): 7 11 12

- Population based screening;
- Insertion of the HPV DNA test as a primary test;
- Refer women positive for HPV-16/18 directly to colposcopy;
- HPV DNA self-sampling for women who reject the professional exam or live in remote areas.

## What diseases are caused by HPV?

### Condyloma acuminata

- What is condyloma acuminata?
They are exophytic lesions caused by HPV, mainly types 6 and 11. They can be painful and/or itchy. In women, they are located in the vulva, perineum, perianal region, vagina and cervix. Less often they develop in extragenital areas, such as nasal and oral mucosa. 13
- How is the diagnosis of condyloma? Clinical diagnosis; biopsy is only indicated in the following cases:
  - Doubt in the diagnosis or suspicion of neoplasia;
  - Lack of response to conventional treatment;
  - When located in the transformation zone of the cervix or anal canal;
  - Immunocompromised patients.
- What are the therapeutic modalities?
They can be self-administered, outpatient or associated. 14

### Self-administered treatments

**Imiquimod cream 5%:** triggers local cellular immune response. Apply to the vulva at night, three times a week on alternate days, washing the area after six to ten hours. It can be used for up to 16 weeks, with an average response in eight weeks. 15 Local reactions such as erythema, itching and burning can occur and are due to the immune response. Systemic adverse effects are rare.

**Podophyllotoxin cream 0.15%:** has antimitotic action. Self-application twice a day for three consecutive days, followed by a four-day pause, repeated weekly for four weeks. 13 Its commercialization was discontinued in Brazil. Purchase by direct import is available.

**5-fluorouracil 5%:** for vulvar lesions, biweekly application on lesions and removal after 4 hours is recommended. There may be erythema, burning and itching. Treatment in the vagina with application of 2.5g weekly or biweekly should be restricted to selected cases with strict monitoring. Indiscriminate use in the past has caused ulcers and vaginal adenosis. 16

### Outpatient treatments

**80%-90% Trichloroacetic Acid (TCA), solution:** promotes chemical coagulation of the condyloma protein content. Apply a small amount to lesions at weekly intervals for eight to ten weeks. A burning sensation occurs at the time of application.

**Podophyllin 10%-25% solution:** vegetable resin with cytotoxic action that inhibits cell metaphase. Apply weekly on each wart, washing after four hours; it is a good option in keratinized lesions. Use must be careful because it is neurotoxic and nephrotoxic.

**Cryotherapy:** promotes thermo-induced cytolysis with liquid nitrogen. It is useful in keratinized lesions. Side effects include pain, erythema and blisters on the spot.

**CO _2_ laser:** promotes tissue evaporation with little deleterious effect. The advantage is the aesthetic and functional result. When epithelial planes are not respected, hypochromia, retraction and alopecia of the treated area may occur. Disadvantages include the cost of equipment and the need for professional training. 16

**Exeresis:** lesions can be removed with a scalpel, scissors or even with electrocautery. There is a risk of scarring and retraction. 14

Podophyllin, 5-fluorouracil and imiquimod should not be used during pregnancy; trichloroacetic acid, laser or cryotherapy are good options. In case of massive lesions, electrocoagulation or tangential exeresis (shaving) are alternatives. There is no indication of cesarean delivery due to the presence of lesions, except in obstruction of the birth canal or bleeding. 13 Recommendations are the same for immunocompromised patients, although closer monitoring is required. Therapeutic options should be changed if there is no significant improvement after three to four weeks of treatment or if lesions remain after six to eight sessions. 13 The treatment of anogenital warts does not eliminate the virus, so lesions can reappear. Infected persons and their partners should return to service if new lesions are identified.

## Low-grade squamous intraepithelial lesion (LSIL)

### What is LSIL?

The natural history of cervical cancer involves cellular changes that can lead to invasive carcinoma. 17 Cytological screening is based on this sequence of changes. 18 LSILs can progress to HSIL and invasive cancer or they can regress to a normal state. 19

LSIL corresponds to the cytological manifestation of HPV infection and has a high potential for regression. 7 Atypical squamous cells of undetermined significance (ASC-US), possibly non-neoplastic ones are characterized by the presence of insufficient cellular alterations for the diagnosis of intraepithelial lesions, although they are more significant than those found in inflammatory processes. 19 These two cytological categories have very similar risks of progressing to HSIL and can be managed in a similar way. 20 21

On the other hand, the reliability of oncotic cytology is important, since the diagnosed LSIL can be associated with the presence of histological HSIL, demonstrating that most of these lesions already existed previously and did not correspond to the evolution of less severe lesions. 20 21 22

The advent of HPV DNA testing allowed risk stratification in patients with LSIL/ASC-US. 17 18 Detection of high-risk HPV DNA in LSIL is associated with a higher risk of progression to HSIL. 21 22 23 Studies have already demonstrated the significant benefits of using the HPV DNA testing in dubious cytologies. 20 21 23 24 25

### How should we conduct LSIL?

Recommendations for patients with a cytopathological diagnosis of LSIL/ASC-US range from immediate referral to colposcopy, repeat cytology or HPV DNA testing with referral to colposcopy if the result is positive. 7 19 20 21 22 23 24 25

Several protocols recommend similar approaches for the cytopathological diagnosis of LSIL and ASC-US: 7 20

- <25 years of age: repeat cytology in three years;
- 25-29 years of age: repeat the cytology in 12 months;
- ≥30 years of age: repeat the cytology in six months;
- Treat infectious processes or atrophy;
- If the repeat cytology is negative in two consecutive exams, return to routine screening;
- If one of subsequent cytologies is positive, perform colposcopy;
- Indicate colposcopy for immunocompromised women with cytopathological examination showing LSIL.

In a recent protocol (2019), the(American Society for Colposcopy and Cervical Pathology (ASCCP) recommended that patients under 25 years of age presenting LSIL cytology, HPV-positive ASC-US or ASC-US without HPV DNA test should repeat isolated cytology one and two years after the initial abnormal result. 21

In patients with ASC-US and negative HPV DNA test, repeat the cytology in three years. After two negative cytological tests, return to routine age-based screening. 20 21

For patients ≥ 25 years of age, if the initial colposcopy is compatible with low grade (histological LSIL/ASC-US/cytological LSIL), repeat cytology and colposcopy in one year. In addition, risk estimates should be used: 20

- HPV DNA standard;
- Patient's age;
- Immunosuppression;
- Changed results of previous cytology and/or biopsy. 20

For patients aged 25 years or older with histology of LSIL diagnosed in consecutive visits for up to two years, observation is preferred, but treatment is acceptable. If treatment is chosen and SCJ and limits of lesions are completely displayed in colposcopic examination, excision or ablation treatments are acceptable. Considering that the biological behavior of smears corresponding to ASC-US/HPV + and LSIL are similar, the approach can be the same. 5 Since regression rates are high and the diagnosis of HSIL in these women is uncommon, continued observation is recommended for a two-year period. 20

## High-grade squamous intraepithelial lesion (HSIL)

### What is HSIL?

The importance of HSIL diagnosis is based on the fact that 70-75% of women with this result will have diagnostic histopathological confirmation of 1-2% of invasive carcinoma. 26 The prevalence of HSIL in cytopathology was 0.26% of tests performed and 9.1% of all tests changed in 2013 in Brazil. 26 27 The term HSIL encompasses cervical intraepithelial neoplasia grades 2 and 3 (CIN 2 and CIN 3) and according to the Lower Anogenital Squamous Terminology (LAST), it can be stratified using the p16 immunohistochemical study, especially in cases of CIN 2 below 30 years of age. 20 28

### How should we conduct HSIL?

The diagnosis of HSIL is made through cytology; referral to colposcopy is mandatory and cytological examination should not be repeated. Biopsy is indicated in the presence of major or discrepant colposcopic findings. 7

When there is cytocolposcopic agreement (HSIL cytology and major findings) in non-pregnant patients over 25 years of age with visible transformation zone (types 1 or 2), without suspicion of invasion or glandular disease, the “See-and-Treat” procedure is indicated. This method avoids unnecessary returns and reduces costs. 7

When the lesion is not fully visible, try to expose the SCJ with a good examination of the vagina to exclude the presence of lesions and indicate excision of the type 3 transformation zone (EZT) with a length of 1.5-2.5 cm. 7 The high-risk HPV DNA testing can be used in the disagreement of methods, with a recognized high negative predictive value. 10

The main objective of excisional treatment is to rule out stromal invasion and assess the state of the surgical margins. 29 30 Therefore, excision should be preferred over ablation. Treatment prevents progression to cancer. Destructive treatment is accepted in selected cases of young patients with a small lesion, ectocervix fully visualized and without suspected invasive disease. 20 Pregnant women with HSIL should be referred to colposcopy to rule out invasion and redo the exams three months after delivery.

Post-treatment follow-up should be done with the HPV-based test (HPV DNA or co-test) six months after the procedure and then annually, until there are three consecutive negative tests. If one of the tests is positive, colposcopy is indicated. If the margins are positive with HSIL (CIN 2+), patient is older than 25 years and formed offspring, repeated excision or observation are acceptable. If recurrent histological HSIL (CIN 2+) is identified and another transformation zone excision does not present technical conditions or is not desired, hysterectomy is recommended. 20 Another option is to follow up with semiannual cytology and colposcopy for two years and annual cytology for five years in the case of compromised margins, when HPV DNA testing is unavailable. 7 Continuous three-year surveillance should be recommended for 25 years, even if patient is over 65 years of age, as the risk of cancer remains twice as high and seems to increase after 50 years of age. 20 Immunosuppressed women are treated like immunocompetent women, with annual post-treatment follow-up throughout their lives, due to the higher risk of recurrences.

## Final considerations

HPV infection is universal in the female genital tract, and can compromise both skin and mucous membranes, causing a series of important nosological manifestations, including genital warts, intraepithelial neoplasms and cancers. Broad coverage of the population through organized screening and vaccination could substantially decrease HPV-induced diseases. The World Health Organization issued a call in 2018 for the elimination of cervical cancer as a serious public health problem (Cervical Cancer Elimination Modeling Consortium - CCEMC): primary prevention strategies, such as the HPV vaccine, and secondary prevention strategies, such as screening, should be strengthened in the coming years. By 2030, the goals are: 90% of girls vaccinated by the age of 15, 70% of women screened with a high-effectiveness test at 35 and 45 years of age; 90% of precursor lesions and invasive cancer treated. Febrasgo, through its National Specialized Commissions on the Lower Genital Tract, Oncology and Vaccines endorses and supports the call.

National Specialty Commission for Lower Genital Tract of the Brazilian Federation of Gynecology and Obstetrics Associations (FEBRASGO)

President:

Neila Maria de Gois Speck

Vice-President:

Márcia Fuzaro Terra Cardial

Secretary:

Mila de Moura Behar Pontremoli Salcedo

Members:

Adriana Bittencourt Campaner

Ana Katherine da Silveira Goncalves

Cláudia Márcia de Azevedo Jacyntho

Fernanda Kesselring Tso

Gustavo Rubino de Azevedo Fochi

Isabel Cristina Chulvis do Val Guimarães

José Humberto Belmino Chaves

Neide Aparecida Tosato Boldrini

Raquel Autran Coelho Peixoto

Rita Maira Zanine

Silvana Maria Quintana

Yara Lucia Mendes Furtado de Melo
